# Immune checkpoint inhibitor-associated toxicity in advanced non-small cell lung cancer: An updated understanding of risk factors

**DOI:** 10.3389/fimmu.2023.1094414

**Published:** 2023-03-06

**Authors:** Xiangxiao Hu, Lina Wang, Bin Shang, Junren Wang, Jian Sun, Bin Liang, Lili Su, Wenjie You, Shujuan Jiang

**Affiliations:** ^1^ Department of Respiratory and Critical Care Medicine, Shandong Provincial Hospital affiliated to Shandong First Medical University, Jinan, China; ^2^ Shandong Key Laboratory of Infectious Respiratory Disease, Jinan, China; ^3^ Medical Science and Technology Innovation Center, Shandong First Medical University & Shandong Academy of Medical Sciences, Jinan, China; ^4^ Department of Respiratory and Critical Care Medicine, Shanghai Ninth People's Hospital, School of Medicine, Shanghai Jiao Tong University, Shanghai, China; ^5^ Department of Thoracic Surgery, Shandong Provincial Hospital Affiliated to Shandong First Medical University, Jinan, China; ^6^ Department of Thoracic Surgery, Fudan University Shanghai Cancer Center, Shanghai, China

**Keywords:** non-small cell lung cancer, immune-related toxicity, combined immunotherapies, predictive biomarker, immune checkpoint inhibitor

## Abstract

Immune checkpoint inhibitors (ICIs), such as programmed death-1 (PD-1), programmed death-ligand 1 (PD-L1), cytotoxic T lymphocyte antigen 4 (CTLA-4) antibodies, etc, have revolutionized cancer treatment strategies, including non-small cell lung cancer (NSCLC). While these immunotherapy agents have achieved durable clinical benefits in a subset of NSCLC patients, they bring in a variety of immune-related adverse events (irAEs), which involve cardiac, pulmonary, gastrointestinal, endocrine and dermatologic system damage, ranging from mild to life-threatening. Thus, there is an urgent need to better understand the occurrence of irAEs and predict patients who are susceptible to those toxicities. Herein, we provide a comprehensive review of what is updated about the clinical manifestations, mechanisms, predictive biomarkers and management of ICI-associated toxicity in NSCLC. In addition, this review also provides perspective directions for future research of NSCLC-related irAEs.

## 1 Introduction

Checkpoint inhibitor-based immunotherapy has changed the landscape of cancer treatment in the past decade. Unlike other treatments, immune checkpoint inhibitors (ICIs) are monoclonal antibodies designed to block negative regulators of T-cell function, including programmed death-1 (PD-1), PD-ligand 1 (PD-L1) and cytotoxic T lymphocyte antigen 4 (CTLA-4), etc, to activate anti-tumor immunity (1). These ICI agents have improved the clinical outcomes in a subset of advanced NSCLC patients. For example, in a study of patients with advanced NSCLC who had received chemotherapy plus concurrent radiation therapy, progression-free survival (PFS) was 16.8 months in patients treated with durvalumab compared to 5.6 months in the placebo group (2). Patients with advanced NSCLC have a 5-fold increase in overall survival (OS) after nivolumab treatment compared to the chemotherapy group, with no severe adverse events (3). Similarly, ipilimumab showed a better potential in extending PFS in NSCLC (4). Specifically, there are seven US Food and Drug Administration (FDA)-approved ICIs for the first-line or second-line treatment of NSCLC, including pembrolizumab, nivolumab, cemiplimab, atezolizumab, durvalumab, tremelimumab and ipilimumab (5–7).

Despite the durable anti-tumor responses, those ICIs also causes many complications in normal tissues, which are referred to as immune-related adverse events (irAEs). Clinical trial data and literatures suggest that irAEs in NSCLC immunotherapy mainly involve the cardiac, pulmonary, gastrointestinal, endocrine, dermatologic and nervous system damage (8–11). While the majority of cases are mild and tolerable, some of them can present with moderate to severe toxicities, which are related to decreased organic function, as well as poor quality of life (12–14). A study showed that the overall incidence of irAEs of any grade in patients with NSCLC was 30%, with a severe grade of 6% (15). However, the clinical features of some early-onset, low-grade immunotoxicity are not easily identified, and it is difficult to determine whether they are associated with ICI therapy. In addition, the detailed mechanisms of irAEs have not been fully elucidated. Therefore, close attention must be paid to better understand the occurrence of irAEs and to screen populations who are most likely to experience irAEs, in order to reduce the toxic effects of immunotherapy. In this review, we will summarize the clinical manifestations of irAEs according to different ICIs and explore possible mechanisms and potential predictive biomarkers of irAEs in NSCLC immunotherapy. At last, we also provide a comprehensive review of current therapies for irAE treatment in NSCLC. This article may have implications for future research.

## 2 Clinical manifestations of irAEs in NSCLC immunotherapy

CTLA-4 and PD-1 are immune checkpoints capable of downregulating T-cell immune functions, but their roles are fundamentally different. Both CTLA-4 and PD-1 have been shown to suppress T cells by blocking CD28-mediated cellular metabolism (16). However, CTLA-4 downregulates T-cell proliferation in the early phase of immune responses, while PD-1 is thought to suppress T-cell immune functions primarily in peripheral tissues in the late stage of immune responses (17). ICI-associated toxicities in NSCLC immunotherapy involve multiple organs throughout the body (**Table 1**). Despite that commonalities in immunotoxicity exist among ICIs, there are differences in specific irAEs, organs involved, as well as clinical manifestations (87).

**Table 1:** Clinical manifestations of common irAEs.

Organ/System	IrAEs	Anti-CTLA-4	Anti-PD-1	Anti-PD-L1	Other ICIs	Combined immunotherapies
**Cardiac**	**Myocarditis**		<1% myocarditis after pembrolizumab (18, 19)	1%-6.7% myocarditis after atezolizumab (20, 21)		1.5% myocarditis after enoblituzumab (anti-B7-H3) plus pembrolizumab (22)
						≤1% myocarditis after ipilimumab plus nivolumab (23, 24)
			<1% myocarditis after cemiplimab (25)			
	**Pericarditis**			2.6% pericarditis after atezolizumab (26)		
	**Pericardial effusion**		2% pericardial effusion after nivolumab (27)	0.4% pericardial effusion after durvalumab (2)		
	**Arrhythmia**	1.7% atrial fibrillation after tremelimumab (28)	<1% atrial tachycardia after cemiplimab (29)	0.2% arrhythmia supraventricular after durvalumab (2)		3% atrial fibrillation after durvalumab plus tremelimumab (30)
		1.7% supraventricular tachycardia after tremelimumab (28)		0.8%-4% atrial fibrillation after durvalumab (2, 31)		
		8% atrial fibrillation after ipilimumab (32)				
	**Heart failure**		2% heart failure after nivolumab (33)	1% heart failure after durvalumab (2)		1% heart failure after ipilimumab plus nivolumab (23)
			<1% heart failure after cemiplimab (25)			
	**Cardiac arrest**					1% cardiac arrest after ipilimumab plus nivolumab (23)
**Pulmonary**	**Pneumonia**	1%-2% pneumonia after tremelimumab (28)	1%-15% pneumonia after cemiplimab (5, 25, 29, 34)	1.6%-16.7% pneumonia after durvalumab (28, 35, 36)	18% pneumonia after vibostolimab (anti-TIGIT) (37)	2% pneumonia after tiragolumab (anti-TIGIT) plus atezolizumab (38)
		2% pneumonia after ipilimumab (39)	3.6%-13% pneumonia after nivolumab (3, 27, 33, 40–42)	5%-30% pneumonia after atezolizumab (43–46)		6% pneumonia after vibostolimab (anti-TIGIT) plus pembrolizumab (37)
			4.4%-23% pneumonia after pembrolizumab (47, 48)	1%-2.3% pneumonia after avelumab (49–51)		16.9% pneumonia after monalizumab (anti-NKG2A) plus durvalumab (35)
						3%-7% pneumonia after ipilimumab plus nivolumab (23, 24)
						7%-12.1% pneumonia after ipilimumab plus pembrolizumab (52, 53)
						0.6%-9% pneumonia after durvalumab plus tremelimumab (28, 54)
	**Pleural effusion**		<2% pleural effusion after cemiplimab (25)			
	**Interstitial lung Disease**	1% interstitial lung disease after ipilimumab (39)	1-3% interstitial lung disease after nivolumab (27, 55)	1% interstitial lung disease after atezolizumab (20)		1% -2% interstitial lung disease after ipilimumab plus nivolumab (24, 56, 57)
				1%-1.3% interstitial lung disease after avelumab (49, 51)		
	**Chronic obstructive pulmonary disease (COPD)**			2.6% COPD after atezolizumab (5)		0.6% COPD after durvalumab plus tremelimumab (28)
				1% COPD after avelumab (49)		
				1.1% COPD after durvalumab (2)		
	**Pneumocystis pneumonia**			1.6% pneumocystis pneumonia after durvalumab (28)		
**Gastrointestinal**	**Nausea/Vomiting**	8%-18% nausea/vomiting after ipilimumab (39, 58)	2%-17.2% nausea/vomiting after pembrolizumab (18, 19)	7.7%-14.2% nausea/vomiting after atezolizumab (26, 59)	15% nausea/vomiting after vibostolimab (anti-TIGIT) (37)	42.9% nausea/vomiting after cobolimab (anti-TIM-3) plus nivolumab (60)
		10% nausea/vomiting after tremelimumab (28)	5%-17% nausea/vomiting after nivolumab (3, 61, 62)	8.7% nausea after cobolimab (60)		1.2%-18% nausea/vomiting after durvalumab plus tremelimumab (28, 63)
			3% nausea/vomiting after cemiplimab (25)	5% nausea after avelumab (49)		
	**Diarrhea**	27%-30% diarrhea after ipilimumab (39, 58)	8.9%-24% diarrhea after nivolumab (3, 21, 40, 41, 61)	0.5%-7% diarrhea after avelumab (49, 51, 64)		57.1% diarrhea after cobolimab (anti-TIM-3) plus nivolumab (60)
		28.4%-41% diarrhea after tremelimumab (28, 65)	48% diarrhea after pembrolizumab (66)	6.2%-20.6% diarrhea after atezolizumab (44, 46, 59, 67)		25% diarrhea after eftilagimod (anti-LAG-3) plus pembrolizumab (68)
			5%-24% diarrhea after cemiplimab (25, 29, 34)	4%-20% diarrhea after durvalumab (2, 28, 31, 36)		11.9% diarrhea after monalizumab (anti-NKG2A) plus durvalumab (35)
						6.4%-20% diarrhea after ipilimumab plus nivolumab (24, 69, 70)
						10%-26% colitis after durvalumab plus tremelimumab (28, 63, 71)
	**Colitis**	8.8%-19% colitis after tremelimumab (28, 65)	2%-4% colitis after nivolumab (27, 41)	2.1% colitis after atezolizumab (44)	3% colitis after vibostolimab (anti-TIGIT) (37)	4% colitis after tiragolumab (anti-TIGIT) plus atezolizumab (38)
		4% colitis after ipilimumab (39)	1%-3.9% colitis after pembrolizumab (18, 72, 73)	0.3%-0.6% colitis after avelumab (51, 64)		1%-6% colitis after ipilimumab plus nivolumab (24, 57)
			<4% colitis after cemiplimab (25)	1.6%-4% colitis after durvalumab (28, 31)		1.8% colitis after durvalumab plus tremelimumab (28)
	**Hepatic injury**	8.3%-30% hepatic injury after tremelimumab (28, 65)	2%-10% hepatic injury after nivolumab (27, 42, 55, 74)	1%-23% hepatic injury after atezolizumab (20, 26, 67)		5% hepatic injury after tiragolumab (anti-TIGIT) plus atezolizumab (38)
		5%-42% hepatic injury after ipilimumab (39, 58)	0.6%-2.1% hepatic injury after pembrolizumab (19, 72, 73, 75)	0.8%-2% hepatic injury after avelumab (49, 51)		11.3%-12.8% hepatic injury after ipilimumab plus pembrolizumab (52)
			<2% hepatic injury after cemiplimab (25)	13% hepatic injury after durvalumab (36)		1%-6% hepatic injury ipilimumab plus nivolumab (23, 24, 76)
						3.5%-9% hepatic injury durvalumab plus tremelimumab (28, 54)
	**Autoimmune hepatitis**		2% autoimmune hepatitis after pembrolizumab (77)	2% autoimmune hepatitis after durvalumab (36)		1.7% autoimmune hepatitis durvalumab plus tremelimumab (28)
			<2% autoimmune hepatitis after cemiplimab (25)			3% autoimmune hepatitis after eftilagimod (anti-LAG-3) plus pembrolizumab (68)
						<1% autoimmune hepatitis after ipilimumab plus nivolumab (24)
	**Pancreatic toxicity**	1.5% pancreatitis after ipilimumab (78)	0.5%-0.6% lipase elevation after nivolumab (3, 18, 27, 55)	12.1% amylase elevation after durvalumab (35)		6.8% amylase elevation after monalizumab (anti-NKG2A) plus durvalumab (35)
		7% pancreatitis after tremelimumab (65)	1%-6% amylase elevation after nivolumab (27, 55, 62)	2% lipase elevation after durvalumab (36)		2%-10.4% pancreatic toxicity after ipilimumab plus nivolumab (53, 79)
			<2% lipase elevation and <4% amylase elevation after cemiplimab (25)	2%-3% amylase/lipase elevation after avelumab (49)		1.2% pancreatitis after durvalumab plus tremelimumab (28)
			<1.2% pancreatitis after pembrolizumab (19, 75, 77)	0.5%-1.3% pancreatitis after atezolizumab (46)		
**Endocrine**	**Hypothyroidism**	4% hypothyroidism after tremelimumab (65)	4%-7.7% hypothyroidism after nivolumab (33, 42, 56, 80)	10.5%-15.2% hypothyroidism after durvalumab (2, 35)		10% hypothyroidism after tiragolumab (anti-TIGIT) plus atezolizumab (38)
			6.7%-28.8% hypothyroidism after pembrolizumab (18, 19, 72, 73, 75, 81)	5.1%-9% hypothyroidism after avelumab (51, 64)		15.3% hypothyroidism after monalizumab (anti-NKG2A) plus durvalumab (35)
			10% hypothyroidism after cemiplimab (29)	5%-14.2% hypothyroidism after atezolizumab (20, 44, 46)		11%-16% hypothyroidism after ipilimumab plus nivolumab (56, 69, 76)
						15.2%-22% hypothyroidism after ipilimumab plus pembrolizumab (52, 53)
	**Hyperthyroidism**	2% hyperthyroidism after ipilimumab (39)	3%-7% hyperthyroidism after nivolumab (42, 82)	2.8%-4.1% hyperthyroidism after atezolizumab (46)		10.2% hyperthyroidism after monalizumab (anti-NKG2A) plus durvalumab (35)
			4%-11.1% hyperthyroidism after pembrolizumab (18, 19, 72, 73, 75, 81)	0.6%-1.3% hyperthyroidism after avelumab (51, 64)		8.7% hyperthyroidism after ipilimumab plus nivolumab (56)
				6.3%-12.1% hyperthyroidism after durvalumab (2, 35, 36)		
	**Thyroiditis**		1%-2% thyroiditis after pembrolizumab (18, 77)	0.5% autoimmune thyroiditis after avelumab (51)		
	**Hypophysitis**	<1% hypophysitis after ipilimumab (39)	0.4%-1% hypophysitis after nivolumab (42, 82)			<1%-2.1% hypophysitis after ipilimumab plus nivolumab (24, 56, 76)
			0.7%-2.4% hypophysitis after pembrolizumab (19, 72, 75, 77)			0.6% hypophysis after durvalumab plus tremelimumab (28)
	**Adrenal insufficiency**	1%-23% adrenal insufficiency after ipilimumab (32, 39)	<3% adrenal insufficiency after nivolumab (27, 62, 83)	1% adrenal insufficiency after atezolizumab (46)		0.6% adrenal insufficiency after durvalumab plus tremelimumab (28)
			0.2%-0.7% adrenal insufficiency after pembrolizumab (19, 75)	0.5%-1% adrenal insufficiency after avelumab (49, 51)		1%-4.7% adrenal insufficiency after ipilimumab plus nivolumab (24, 56)
			1% adrenal insufficiency after cemiplimab (29)	2% adrenal insufficiency after durvalumab (36)		2% adrenal insufficiency after ipilimumab plus pembrolizumab (53)
	**Diabetes/ Hyperglycemia**		0.2%-0.6% diabetes after pembrolizumab (73, 75)	3% hyperglycemia after durvalumab (35)		4% diabetes after tiragolumab (anti-TIGIT) plus atezolizumab (38)
			2%-5% hyperglycemia after nivolumab (33, 55)			10.2% hyperglycemia after monalizumab (anti-NKG2A) plus durvalumab (35)
						2% diabetes after ipilimumab plus pembrolizumab (53)
						1% hyperglycemia after ipilimumab plus nivolumab (23)
**Dermatologic**	**Vitiligo**		1% Vitiligo after nivolumab (82)			
	**Rash**	17%-28% rash after ipilimumab (39, 58, 84)	5.7%-27% rash after nivolumab (27, 40, 42, 55)	5%-11% rash after atezolizumab (20, 44, 45)	24% rash after vibostolimab (anti-TIGIT) (37)	27% rash after tiragolumab (anti-TIGIT) plus atezolizumab (38)
		13.3%-41% rash after tremelimumab (28, 65)	7%-22% rash after pembrolizumab (18, 19, 73, 77)	3.8% rash after avelumab (64)		21% rash after vibostolimab (anti-TIGIT) plus pembrolizumab (37)
			5% rash after cemiplimab (25)	3.2%-15% rash after durvalumab (28, 35, 36)		14.5% rash after cobolimab (anti-TIM-3) plus dostarlimab (anti-PD-1) (60)
						10.4%-20% rash after ipilimumab plus nivolumab (24, 56, 69, 76)
						11.3% rash after enoblituzumab (anti-B7-H3) plus pembrolizumab (22)
						7% rash after durvalumab plus tremelimumab (28, 71)
	**Nodular eczema**		0.2% nodular eczema after nivolumab (3)			
	**Erythema multiforme**	11% erythema multiforme after tremelimumab (65)	2.1%-7% erythema multiforme after nivolumab (41, 42, 62)			
			2% erythema multiforme after cemiplimab (25)			
	**Dermatitis acneiform**		0.5% dermatitis acneiform after nivolumab (56)	4.8% dermatitis acneiform after durvalumab (28)		1.2% Dermatitis acneiform after ipilimumab plus nivolumab (56)
				0.3% dermatitis acneiform after avelumab (51)		
	**Acneform eruptions**		5.4% acneform eruptions after nivolumab (83)			
	**Maculopapular Rash**		6.3%-15% maculopapular rash after nivolumab (41, 83)	2% maculopapular rash after atezolizumab (45)		6% maculopapular rash after durvalumab plus tremelimumab (54)
			6% maculopapular rash after cemiplimab (29)	0.8%-1.3% maculopapular rash after avelumab (51, 64)		8% maculopapular rash after tiragolumab (anti-TIGIT) plus atezolizumab (38)
						5%-14% maculopapular rash after ipilimumab plus nivolumab (56, 57, 85)
	**Herpes**		<2% herpes zoster after nivolumab (27)			
			2% pemphigoid after nivolumab (62)			
	**Dermatitis**			1.1% dermatitis after durvalumab (2)		
	**Psoriasis**		4% Psoriasis after nivolumab (80)	1% Psoriasis after avelumab (49)		
**Musculoskeletal System**	**Arthralgia**	7%-14% arthralgia after ipilimumab (39, 58)	5.7%-26% arthralgia after nivolumab (3, 41, 62)	2%-16.8% arthralgia after atezolizumab (20, 21, 59, 67)	18% arthralgia after vibostolimab (anti-TIGIT) (37)	16% arthralgia after tiragolumab (anti-TIGIT) plus atezolizumab (38)
			4%-20.5% arthralgia after pembrolizumab (18, 72)	5% arthralgia after avelumab (49)		12% arthralgia after vibostolimab (anti-TIGIT) plus pembrolizumab (37)
			4%-13%arthralgia after cemiplimab (25, 29)	16.7% arthralgia after durvalumab (35)		7%-9.9% arthralgia after ipilimumab plus pembrolizumab (52, 53)
	**Arthritis**			1%-4% arthritis after avelumab (36, 49)		
	**Joint effusion**		<1% joint effusion after nivolumab (27)			
	**Myalgia**	7% myalgia after ipilimumab (39)	4%-20% myalgia after nivolumab (27, 40, 41, 62)	13.5% myalgia after atezolizumab (44)	6% myalgia after vibostolimab(anti-TIGIT) (37)	3% myalgia after vibostolimab (anti-TIGIT) plus pembrolizumab (37)
			3% myalgia after pembrolizumab (18)			7% myalgia after ipilimumab plus pembrolizumab (53)
	**Myositis**		<1%-4% myositis after nivolumab (27, 80)			
			0.5%-2% myositis after pembrolizumab (19, 73, 77)			
	**Myasthenia**		<1% myasthenia syndrome after nivolumab (27)			2% myasthenia syndrome after ipilimumab plus pembrolizumab (53)
			1% myasthenia gravis after nivolumab (82)			4.3% myasthenia gravis after atezolizumab plus ipilimumab (86)
						1.8% myasthenia gravis after durvalumab plus tremelimumab (28)
**Kidney**	**Elevated creatinine/ Renal injury**	<1% renal injury after ipilimumab (39)	2%-3.2% renal injury after pembrolizumab (73, 77)	0.4% renal injury after durvalumab (2)		1% acute kidney injury after ipilimumab plus pembrolizumab (23)
			1% renal injury after cemiplimab (29)			1% renal injury after ipilimumab plus nivolumab (23)
						0.6%-4% renal injury after durvalumab plus tremelimumab (28, 30)
	**Nephritis**		0.4%-0.6% nephritis after pembrolizumab (19, 73)			
	**Allergic nephritis**		4% allergic nephritis after nivolumab (41)			
	**Renal failure**		5% renal failure after nivolumab (41)			1.2% renal failure after durvalumab plus tremelimumab (28)
**Eyes**	**Uveitis**	1.5% uveitis after ipilimumab (78)	0.6% uveitis after pembrolizumab (73)			9% uveitis after ipilimumab plus pembrolizumab (53)
	**Scleritis**	1% scleritis after ipilimumab (78)		1% scleritis after avelumab (49)		
**Oral cavity**	**Stomatitis**	11.7% stomatitis after tremelimumab (28)	0.6% stomatitis after pembrolizumab (73)	11.9% stomatitis after atezolizumab (59)		
**Nervous system/ Brain**	**Encephalitis**		<1% encephalitis after nivolumab (27)	0.3% encephalitis after avelumab (51)		
	**Peripheral neuropathy**	11%-14% peripheral neuropathy after ipilimumab (39, 58)	20% peripheral neuropathy after nivolumab (41)	38.7% peripheral neuropathy after atezolizumab (44)		
			1%-20.5% peripheral neuropathy after pembrolizumab (18, 72)			
	**Atypical Guillain-Barre syndrome**		4% Atypical Guillain-Barre syndrome after nivolumab (80)			

### 2.1 Anti-CTLA-4

CTLA-4 plays an essential role in attenuating T-cell responses, preventing immune deregulation, inducing immune tolerance, and controlling autoimmunity (88). CTLA-4 is expressed on the surface of activated T cells, while its ligands, CD80/CD86, is predominantly expressed on antigen-presenting cells (APCs), including dendritic cells (DCs) and macrophages. In the early phase of T-cell activation, CTLA-4 competes with CD28 for binding to CD80/CD86 in APCs (89, 90). Additionally, CTLA-4 expressed on regulatory T cells (Tregs) can reduce the availability of CD80/CD86 expressed on DCs through trans-endocytosis (91), and induce the production of broadspectrum, immunosuppressive cytokines, such as transforming growth factor-β (TGF-β) and interleukin-10 (IL-10) (92, 93), leading to an attenuated antigen-presenting activity of APCs and a suppression of T-cell activation. While the lack of CTLA-4 gene can result in multi-organ lymphocytic infiltration, Treg cell defect and autoantibody production, CTLA-4 blockade did not induce substantial autoimmunity (90, 94, 95).

The most frequently reported irAEs in NSCLC patients receiving anti-CTLA-4 therapy were diarrhea, rash and nausea/vomiting (**Table 1**). The incidence of gastrointestinal system adverse events was relatively high, particularly diarrhea, hepatic injury and nausea/vomiting, with the clinical data showing an incidence of approximately 30% for diarrhea, 5%-40% for hepatic impairment, and 4%-8% for enteritis (**Table 1**). And the incidence of skin toxicity is also high where the incidence of rash is about 17%-41% (**Table 1**). As shown in **Table 1**, the incidence of adrenal insufficiency in NSCLC receiving anti-CTLA-4 was significantly higher than that of patients treated with anti-PD-1/PD-L1 drugs. The occurrence of checkpoint inhibitor pneumonitis (CIP) was relatively rare in patients treated with anti-CTLA-4 compared with other ICIs; however, its true incidence may be underestimated (96).

At present, anti-CTLA-4 drugs for the treatment of NSCLC are ipilimumab and tremelimumab; however, there are clear differences with regard to the toxicity of these two drugs in different organs. Clinical trials suggested that common adverse reactions to ipilimumab for NSCLC are diarrhea (27%-30%), rash (17%-28%), nausea/vomiting (8%-18%), hepatic impairment (5%-42%), arthralgia (7%-14%), and pituitary inflammation (1%) (**Table 1**). Moreover, ipilimumab treatment can be associated with atrial fibrillation, uveitis, pancreatic toxicity, and peripheral neuropathy. As for tremelimumab, gastrointestinal and skin toxicity are the most common adverse events in patients with NSCLC, with nausea and vomiting at 10%, diarrhea at 30%, hepatic impairment at 8.3%-30%, and skin rash at 13%-41% (**Table 1**).

### 2.2 Anti-PD-1/PD-L1

PD-1 is expressed on activated T cells, B cells, natural killer (NK) cells, and cells of the myeloid lineage. PD-L1 is one of the ligand which can be expressed on a variety of immune and non-immune cells, including tumor cells, while the expression of another ligand of PD-1, PD-L2, is limited to APCs (97, 98). Compared to CTLA-4, PD-1 is involved in a broader range of immune regulation and mainly suppresses the effector phase of activated T cells (99). The binding of PD-1 to its ligands impairs T-cell proliferation and cytokine production by abrogating the cascade response activity of two signals, namely the PI3K/Akt and Ras/MEK/Erk pathways (16, 100–102). Notably, the binding of PD-1 to different ligands leads to distinct biological effects (103). The binding of PD-1 with PD-L2 leads to an enhanced Th2 activity, whereas PD-1/PD-L1 binding inhibits T-cell responses (104, 105). Anti-PD-1/PD-L1 antibodies are also known as “immune normalizers”, which are intended to “normalize” T-cell immunity in tumor microenvironment (106). IrAEs are manifestations of immune imbalance when anti-PD-1/PD-L1 antibodies enhance T cell-mediated immune responses in normal tissues.

Gastrointestinal toxicity is the most common adverse event in NSCLC receiving anti-PD-1/PD-L1 antibodies, followed by pulmonary, neurological, cutaneous, endocrine and musculoskeletal toxicities (**Table 1**). Clinical data showed that the incidence of gastrointestinal adverse events is the highest, with diarrhea (1%-48%), nausea/vomiting (2%-17.2%) and hepatitis (1%-23%) being the most commonly reported (**Table 1**). This was followed by CIP (1%-30%), and the presence of severe pneumonia can severely compromise the efficacy of ICI therapy (107, 108). The incidence of peripheral nerve injury (1%-38%) is high; however, mild neurological toxicity may be easily overlooked (**Table 1**). In addition, all ICIs have a high incidence of skin toxicity, with anti-PD-1/PD-L1 having the highest incidence of rash (3%-27%) and maculopapular rash (1%-15%) (**Table 1**). Overall, the incidence of major endocrine toxicities associated with anti-PD-1/PD-L1 therapy is generally low, with the exception of hypothyroidism (1%-28%) (**Table 1**). Other adverse events with a relatively high incidence were arthralgia (2%-26%) and muscle pain (3%-20%) (**Table 1**). As reported, diarrhea, rash, maculopapular rash and renal injury were significantly higher in anti-PD-1 than in anti-PD-L1. Conversely, the incidence of myocarditis, stomatitis and peripheral neuropathy were higher in anti-PD-L1 than in anti-PD-1 (**Table 1**).

On the other hand, the incidence of adverse events differs significantly between different anti-PD-1/PD-L1 antibodies. The higher incidence of irAEs with nivolumab in the treatment of NSCLC are rash (5.7%-27%), diarrhea (8.9%-24%), arthralgia (5%-26%), and myalgia (4%-20%) (**Table 1**). Events such as pneumonia, interstitial pneumonia, nausea/vomiting, enteritis, hepatic impairment, hypothyroidism, hyperthyroidism, pituitary inflammation and hyperalgesia were also frequently reported. Hypothyroidism (6.7%-28.8%), pneumonia (4.4%-23%), rash (7%-22%), nausea/vomiting (2%-17.2%) and hyperthyroidism (4%-11%) are the irAEs of a higher incidence with pembrolizumab (**Table 1**). Gastrointestinal toxicity such as diarrhea (5%-24%) was the highest and most frequently reported adverse event in cemiplimab therapy (**Table 1**). Adverse events involving the respiratory and circulatory systems were frequently reported with cemiplimab, but not at a high incidence rate. Adverse events of a high incidence with durvalumab are diarrhea (4%-20%), pneumonia (1.6%-16.7%), hypothyroidism (10.5%-15.2%), rash (3.2%-15%), hepatic impairment (13%), hyperthyroidism (6.3%-12.1%) and pancreatitis (2%-12.1%) (**Table 1**). The frequently reported adverse events with atezolizumab were diarrhea (6.2%-20.6%), hepatic impairment (1%-23%), nausea/vomiting (7.7%-14.2%), hypothyroidism (5%-14.2%), arthralgia (2%-16.8%), myalgia (13.5%) and rash (5%-11%) (**Table 1**). Avelumab had a significantly lower incidence of adverse reactions than durvalumab or atezolizumab, with the commonly reported adverse events being arthralgia (5%) and rash (3.2%) (**Table 1**).

### 2.3 Other ICIs

Recently, the conversion of other immune checkpoints, such as lymphocyte activation gene-3 (LAG-3), T cell immunoglobulin and ITIM domain (TIGIT) and T cell immunoglobulin and mucin-domain containing-3 (TIM-3), B7-H3 and NKG2A, into clinical next-generation immunomodulatory targets has been increasingly investigated (109). The binding of LAG-3 to corresponding ligands, including major histocompatibility complex II (MHC-II), liver sinusoidal endothelial cell lectin (LSECtin) and hepatic fibrinogen-like protein 1 (FGL-1), induces immune cell depletion and reduces cytokine secretion (110–114). On the other hand, LAG-3 deficiency leads to an increased autoimmune susceptibility in mice (115, 116). REGN3767 is a human LAG-3 monoclonal antibody. In clinical trials of REGN3767 monotherapy for advanced malignancies, the most frequent adverse reaction was nausea (22.2%) (117). Whether LAG-3 potentiates irAEs of known checkpoint blockers or generates new irAEs still needs to be further explored. TIGIT is an inhibitory receptor that is widely expressed on lymphocytes (118, 119). The interaction of TIGIT with its ligands (CD155, CD112 and CD113) can impair DC-triggered T-cell responses and inhibit the cytotoxicity of CD8^+^ T cells (120, 121). IrAEs associated with anti-TIGIT drugs (vibostolimab) were rash (24%), pneumonia (18%), arthralgia (18%), nausea/vomiting (15%), myalgia (6%), and enteritis (3%) (**Table 1**). TIM-3 is expressed in tumor cells and immune cells. The interaction of TIM-3 with its ligand induces T-cell suppression (122). TIM-3 is highly upregulated on infiltrating CD4^+^ and CD8^+^ T cells from human lung cancer tissues, and high TIM-3 expression may be associated with tumor progression (123). Phase I clinical trials found that the most frequent adverse events with anti-TIM-3 monotherapy (cobolimab) in advanced solid tumors (NSCLC and melanoma) were fatigue (13.0%) and nausea (8.7%) (60). B7-H3, also called CD276, is a member of the B7 family, which was also recognized as a co-stimulatory molecule. Although its ligand has not been well studied, certain treatments targeting B7-H3 are undergoing clinical trials. Inhibitors of immune checkpoints such as VISTA, ICOS and BTLA are currently in clinical trials for the treatment of advanced solid tumors.

### 2.4 Combined immunotherapies

The most common combination regimens are anti-CTLA-4 plus anti-PD-1/PD-L1, anti-PD-1/PD-L1 plus anti-TIGIT, anti-PD-1 plus anti-B7-H3, anti-PD-L1 plus anti-NKG2A, anti-PD-1 plus anti-LAG-3, anti-PD-1 plus anti-TIM-3, and anti-PD-1 plus anti-B7-H3. Compared with the application of one checkpoint inhibitor or chemotherapy, the combination of ICIs for NSCLC was associated with an increased incidence of adverse reactions and high-grade immunotoxicities (Grade ≥ 3) in patients while prolonging the PFS (23, 79, 124). Gastrointestinal toxicities (10%-50%) and endocrine toxicities (1%-20%) are significantly higher in combined immunotherapy than in monotherapy, such as diarrhea (6.4%-57%), nausea/vomiting (1.2%-42.9%), hypothyroidism (10%-22%), hyperthyroidism (8.7%-10%), hypothyroidism and glucose abnormalities (1%-10.2%) (**Table 1**). Elsewhere, the incidence of maculopapular rash (5%-14%) and uveitis (9%) was relatively higher in combined immunotherapy than in monotherapy (**Table 1**). The differences in cardiac and pulmonary toxicity were not significant (**Table 1**). Adverse events with higher rates in ipilimumab plus nivolumab are rash (10%-20%), diarrhea (6.4%-20%), hypothyroidism (11%-16%), maculopapular rash (5%-14%), pancreatitis (2%-10.4%), hyperthyroidism (8.7%), pneumonia (3%-7%), enterocolitis (1%-6%), hepatic impairment (1%-6%), and hyperaldosteronism (1%-4.7%) (**Table 1**). The incidence of enterocolitis (10%-26%) and arrhythmias (3%) with tremelimumab plus durvalumab treatment was significantly higher than other combination regimens (**Table 1**). The most prominent adverse events with ipilimumab plus pembrolizumab were pneumonia (7%-17.2%) (**Table 1**). In contrast, the overall respiratory, gastrointestinal and endocrine system toxicity was higher with anti-PD-1/PD-L1 in combination with other ICIs for NSCLC than with anti-CTLA-4 plus anti-PD-1/PD-L1 (**Table 1**).

## 3 Mechanism of irAEs

**Figure 1:**
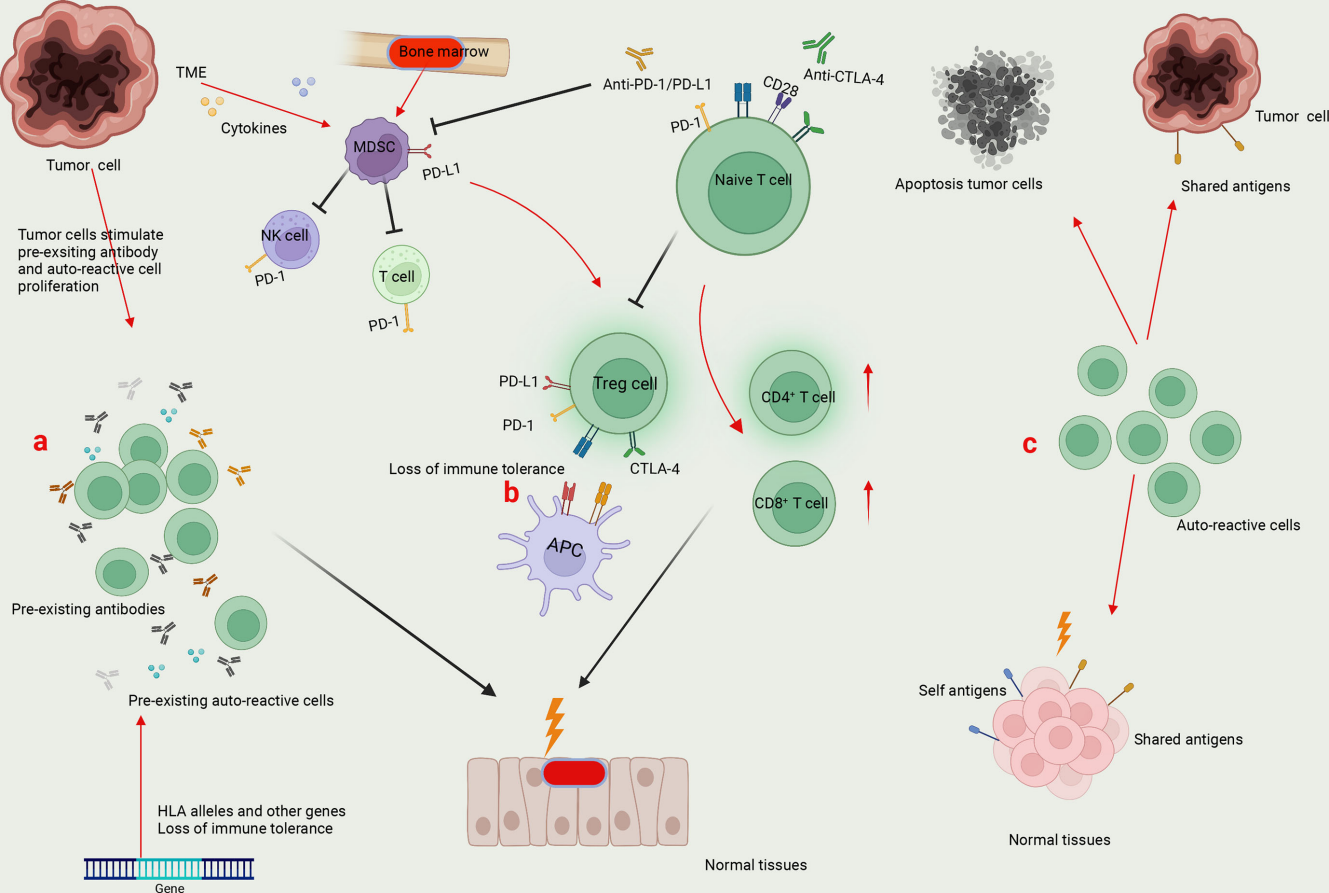
Mechanisms underlying irAEs. **(A)** Specific HLA alleles and other genes result in the generation of pre-existing auto-reactive cells and antibodies in cancer patients. The enhanced auto-reactive cells by checkpoint inhibitors further promotes the production of autoantibodies, leading to the attack of normal tissues. **(B)** Tumor microenvironment (TME) favors the generation of myeloid-derived suppressor cells (MDSCs), which directly suppress antitumor immunity by expressing PD-L1 and indirectly induce Treg differentiation to inhibit T-cell function. On one hand, the application of ICIs to block PD-1/PD-L1 and CTLA-4-mediated immune suppression may affect the function of MDSCs and Tregs, leading to the loss of immune tolerance. On the other hand, by promoting the effector function of CD4^+^ T and CD8^+^ T cells, ICI treatment enhances the potential immune responses against normal tissues. **(C)** Tumor cells can express shared antigens with normal tissues. Meanwhile, tumor cell death releases self and tumor antigens. The auto-reactive T cells can recognize shared or self antigens, leading to the collapse of immune tolerance and normal tissue damage.

The underlying mechanism of irAEs in NSCLC immunotherapy remains largely unknown. Here, we summarized three main factors that may be involved in the pathogenesis of irAEs: pre-existing autoimmunity, loss of tolerance, and presentation of self-antigens (**Figure 1**). Currently, accumulating evidence suggested that genetic predisposition or subclinical autoimmunity contributes to the development of irAEs. Specific HLA alleles may enhance the autoimmune response and be associated with the production of autoantibodies (125, 126) (**Figure 1**). For example, the presence of HLA-DRB1 allele is associated with the specific autoantibody, antithyroglobulin (127, 128). HLA allele DRB1*04:05 is associated with the development of arthritis in ICI-treated patients (129). A case series study revealed that HLA-DR4, a genetic predictor for type 1 diabetes, is enriched in ICI-related diabetes, which can also be considered as a complication of autoimmune pancreatitis induced by ICIs (130). Another study demonstrated that in advanced NSCLC patients with subclinical autoimmune disease, the pre-existing antibodies were positively correlated with the occurrence of irAEs after anti-PD-1 treatment (131). Loss of immune tolerance due to the HLA allele and other genes results in the production of heterogeneous autoantibodies and inflammatory cytokines that may be present for months or even years (**Figure 1**). In response to appropriate environmental factors, auto-reactive cells are activated and recruited to target tissues (128, 132) (**Figure 1**).

Except for activated T cells, CTLA-4 and PD-1 are constitutively expressed by Tregs and some myeloid cells, which play a vital role in maintaining immune tolerance (133, 134) (**Figure 1**). A canonical theory of irAEs is that treatment with ICIs leads to the loss of tolerance in cancer patients, paving ways for auto-reactive T-cell activation and the overwhelming humoral immunity (**Figure 1**). For instance, a previous study dissected a significant accumulation of CD8^+^ T cells with highly cytotoxic and proliferative status in ICI-associated colitis, a substantial fraction of which were found to originate from tissue-resident populations in TCR sequence analysis (135). It has been recognized that the most common irAE tends to occur in organs that are highly dependent on peripheral tolerance to maintain immune homeostasis (136).

Furthermore, tumor cells can express shared antigens with normal tissues, potentializing the excessive T-cell activity upon ICI treatment, which is thought to be another primer factor in the development of irAEs (**Figure 1**). In immune-related skin toxicity in NSCLC patients, nine T-cell shared antigens between tumor and skin tissues were identified (137). In CIP of NSCLC patients, a significant overlap of T-cell repertoire was detected *via* gene sequencing between tumor-infiltrating lymphocytes (TILs) and CIP-infiltrating lymphocytes, rather than T cells from either the secondary lymphoid organs or peripheral blood (138). Consistently, ipilimumab treatment led to a greater diversification of T-cell repertoire, a clear indicator of auto-reactivity to multiple antigens, in cancer patients with irAEs compared with those without irAEs (139). Thus, it has been realized that ICI-induced epitope spreading represents a newly specific mechanism accounting for this broadening of T-cell repertoire and cross-reactivity in cancer patients.

Other factors, such as non-autoimmune inflammation and gut microbiome, may also play a critical role in the development of irAEs (140, 141). Ultimately, as irAEs become a limitation in anti-tumor immunotherapy, more efforts are required in future to learn the pathophysiology of irAEs.

## 4 Predictive biomarkers

**Figure 2:**
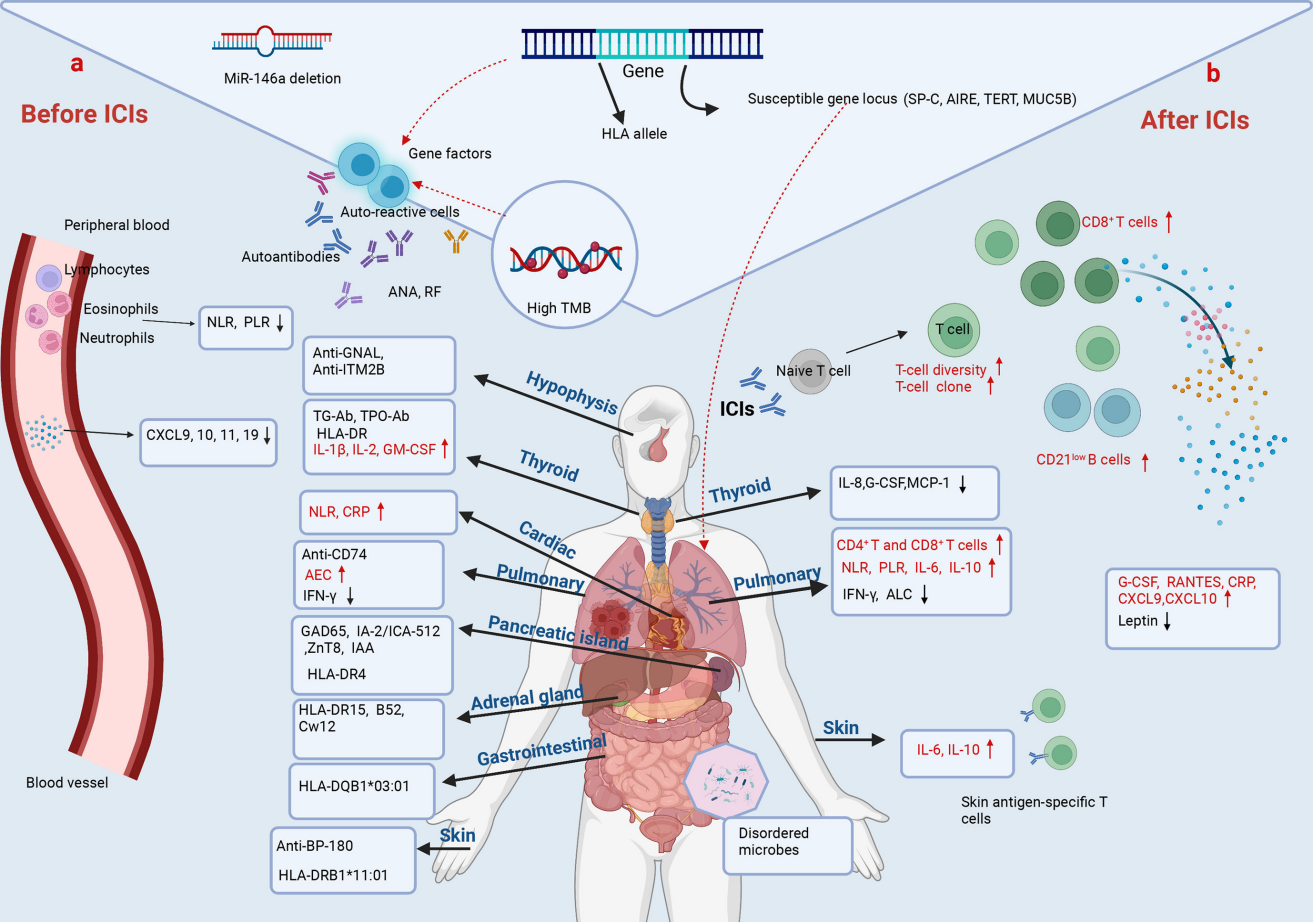
Predictive biomarkers of irAEs in NSCLC. **(A)** Before ICIs. The baseline cytokines, chemokines, peripheral-blood cell counts, and pre-existing antibodies and genetic alterations are associated with irAEs in NSCLC. Lower baseline levels of NLR, PLR, CXCL9, CXCL10, CXCL11 and CXCL19 correlate with irAEs. MiR-146a deletion even predicts the occurrence of severe irAEs. However, the baseline NLR and CRP are increased in NSCLC with immune-related cardiotoxicities. The pre-existing anti-GNAL and anti-ITM2B in plasma predict immune-related hypophysitis. ICI-related thyroid dysfunction is associated with pre-existing TG-Ab, TPO-Ab, HLA-DR, and higher baseline value of IL-1β, IL-2 and GM-CSF. Anti-CD274, a higher baseline level of AEC and lower baseline IFN-γ and ALC are associated with CIP. HLA alleles and autoantibodies as indicated are related to immune-related diabetes, colitis, adrenal and skin adverse events. Age, BMI and past medical history have also been shown to be strongly associated with irAEs. **(B)** After ICIs. An increased diversification of peripheral T-cell repertoire, early expansion of CD8^+^ T-cell clones (≥ 55), and increased circulating CD21^low^ B cells characterized by higher PD-L1 expression and greater clonality after immunotherapy are associated with irAEs. Higher post-treatment levels of serum G-CSF, RANTES, CRP, CXCL9 and CXCL10, and lower post-treatment leptin correlate with irAEs. Changes of cytokines during ICI treatment predict organ-specific adverse events. Decreased IL-8, G-CSF and MCP-1 predict immune-related thyroid dysfunction. Increased IL-6 and IL-10, and decreased IFN-γ predict CIP. Increased IL-6 and IL-8, and the presence of skin antigen-specific T cells in blood after ICIs predict skin toxicities. In addition, disturbances in gut microbes are associated with gastrointestinal toxicities.

In the past few years, biomarkers have been investigated to predict adverse effects during cancer immunotherapy. To date, the potential biomarkers reported mainly include immune cells, cytokines/chemokines, autoantibodies, genetics and other factors (**Figure 2**).

### 4.1 Immune cells

Considering the central role in mediating the toxicities of ICI therapy, immune cells have long been recognized as attractive cellular biomarkers in the prediction of irAEs. Research has focused on using immune signatures in peripheral blood as candidate biomarkers given the ease of access and minimal invasiveness. In agreement with the pathogenesis of irAEs, skin antigen-specific T cells were found in blood samples of anti-PD-1-treated NSCLC patients who developed autoimmune skin toxicities, implying a potential predictive value of this immune cell subset for skin-related irAEs (137) (**Figure 2**). Similarly, an early expansion of CD8^+^ T cell clones (≥ 55) in peripheral blood within two weeks of ICI starting therapy indicates the forthcoming occurrence of severe irAEs in prostate cancers receiving ipilimumab (142) (**Figure 2**). Significant increases in CD4^+^ T and CD8^+^ T cells were observed in biopsies from cancer patients treated with PD-1/PD-L1 inhibitors resulting in mechanical pneumonia, and in bronchoalveolar lavage (BAL) samples from patients with CIP (143, 144) (**Figure 2**). An early and greater diversification of peripheral T-cell repertoire after checkpoint blockade was observed in cancer patients with irAEs than in those without irAEs, indicating its predictive value for irAE (139) (**Figure 2**). Moreover, recent studies also suggested the utility of humoral immune cells in irAE identification. Das et al. reported that an early increase in circulating CD21^low^ B cells, characterized by a higher expression of PD-L1 and a greater clonality than CD21^high^ B subsets, correlated with severe irAEs after anti-PD-1 and anti-CTLA-4 combined therapy (145) (**Figure 2**).

### 4.2 Cytokines and chemokines

Cytokine release syndrome (CRS) is a systemic inflammatory disorder characterized by a massive release of cytokines, which may occur in cancer patients receiving ICIs treatment (146). Since the storm of T cell-activating cytokines induces broad over-activity of T cells targeting self-tissues, a series of symptoms, ranging from mild to life-threating, can accompany with CRS. In accordance, recent studies have evaluated the baseline and changes of cytokines and chemokines in cancer patients to predict adverse reactions of ICIs therapy. In a longitudinal study, higher post-treatment levels of serum G-CSF and RANTES, as well as a lower level of leptin after treatment, were observed in irAE NSCLC patients, compared with non-irAE NSCLC patients (147) (**Figure 2**). Another study, which included 53 lung cancer patients, indicated that the subjects who developed irAEs possessed lower baseline levels of CXCL9, CXCL10, CXCL11 and CXCL19, as well as a greater increase in CXCL9 and CXCL10 at post-treatment, compared with those without irAEs (148) (**Figure 2**).

As for organ-specific irAEs, Lin et al. reported that increased levels of serum IL-6 and IL-10 during ICI treatment may act as useful biomarkers to predict CIP in lung cancer patients (149) (**Figure 2**). Another study of NSCLC even showed that both a low baseline level of IFN-γ and decrease of IFN-γ after ICI therapy were correlated with the development of CIP (150) (**Figure 2**). Additionally, higher baseline levels of IL-1β, IL-2 and GM-CSF, as well as early decrease of IL-8, G-CSF and MCP-1 were demonstrated to be associated with ICI-related thyroid dysfunction (151) (**Figure 2**). Phillips et al. retrospectively reviewed patients with immune-related cutaneous adverse events (ircAEs), and suggested that increased serum IL-6 and IL-10 may act as potential biomarkers for ircAEs (152) (**Figure 2**).

### 4.3 Autoantibodies

Cancer patients with co-existing autoimmune diseases are at a higher risk for the development of irAEs. This notion is supported by the following studies, suggesting that pre-existing autoantibodies in the body of cancer patients are predictive of irAEs following ICI treatment. It has been reported that autoantibodies, including anti-GNAL, anti-ITM2B and anti-CD74, both in pre-treatment and on-treatment plasma samples of cancer patients correlate with the development of immune-related hypophysitis and pneumonitis after ICI therapy, respectively (153) (**Figure 2**). The presence of anti-thyroid peroxidase and anti-thyroglobulin antibodies at baseline were associated with a higher risk of thyroid dysfunction during immunotherapy (154–157) (**Figure 2**). And an elevated anti-BP-180 antibody at baseline correlated with a higher probability to develop skin adverse events in NSCLC during anti-PD-1/PD-L1 therapy (158) (**Figure 2**). In addition, insulin-dependent diabetes occurred in nearly 1% of solid cancer patients treated with anti-PD-1/PD-L1 antibodies, of which 40% had at least one positive auto-antibody and 21% had two or more, including glutamic acid decarboxylase (GAD65), islet antigen2 (IA-2/ICA-512), zinc transporter8 (ZnT8) and insulin autoantibodies (IAA) (159) (**Figure 2**). Toi et al. profiled the pre-existing autoimmune markers in advanced NSCLC patients with subclinical autoimmune disease who underwent anti-PD-1 therapy, and found that the presence of pre-existing antinuclear antibody (ANA), rheumatoid factor (RF), anti-thyroglobulin (TG) and anti-thyroid peroxidase (TPO) were associated with the development of irAEs, as well as with clinical benefits to PD-1 inhibitors (131) (**Figure 2**). Mechanistically, this correlation can be explained that the enhanced auto-reactive T cells can induce the production of auto-antibodies in B cells following ICIs treatment, which eventually cause irAEs (160).

However, not all antibody positivity leads to adverse effects in ICI treatment. A recent study showed that patients with ICI-induced inflammatory arthritis were more likely to be autoantibody-negative for RF and anti-cyclic citrullinated peptide (CCP) antibodies (129). Importantly, it should be noteworthy that cancer patients with pre-existing autoimmune disorders were often excluded from access to immunotherapy-based, anti-cancer clinical trials and only small-scale retrospective cohort studies are currently available to determine the correlation between autoantibodies and irAEs. Thus, further well-designed larger prospective studies are needed to validate these findings and investigate whether a specific autoimmune framework predicts different irAEs.

### 4.4 Genetic alterations

Recently, genetic pre-disposition to irAEs has attracted increasing attention. Hoefsmit et al. reported that susceptible genetic loci of immune-related genes, including surfactant protein C (SP-C), autoimmune regulator (AIRE), telomerase reverse transcriptase (TERT) and mucin 5B oligomeric mucus/gel-forming (MUC5B), are linked to the development of CIP in cancer patients (161) (**Figure 2**). A recent database study reported a positive correlation between tumor mutational burden (TMB) and anti-PD-1-induced irAEs across 18 cancer types (162) (**Figure 2**). In addition, certain HLA types were also found to be associated with the development of specific irAEs. A retrospective study of cancer patients by Danae et al. found an increased expression of HLA-DR on the surface of monocytes in patients with thyroid toxicity caused by anti-PD-1 inhibitor (pembrolizumab) (163) (**Figure 2**). Studies on the treatment of advanced cancer patients including NSCLC by ICIs showed that HLA-DR15, B52, and Cw12 may be related to the occurrence and development of adrenal insufficiency (164) (**Figure 2**). A prospective observational clinical study dissected associations between HLA-DRB1*11:01 and pruritus, and between HLA-DQB1*03:01 and colitis in metastatic NSCLC and melanoma patients receiving anti-PD-1, anti-CTLA4, or both (165) (**Figure 2**). Stamatouli and colleagues identified a predominance of HLA-DR4 in 76% of cancer patients with autoimmune, insulin-dependent diabetes caused by ICIs, suggesting the potential of HLA-DR4 in identifying those at a higher risk to develop this kind of irAE (159) (**Figure 2**). In addition, preclinical data showed that microRNA-146a (miR-146a) deletion was associated with the occurrence of severe irAE (166) (**Figure 2**). However, despite this progress, the predictive value of genetic alterations for irAEs remains less well-defined. Larger association studies are required to extend the usage of genetic determinants in irAEs.

### 4.5 Other factors

Even more creative strategies to predict irAEs have been considered. Until now, there are some other emerging irAE biomarkers on the rise, such as demographic information, body mass index (BMI), past medical history, peripheral-blood cell count, gut microbiome and omics information. A retrospective study of cancer patients receiving pembrolizumab treatment found that an increase in BMI was associated with an increased risk of irAEs (167). It has been reported that patients older than 70 years or with a smoking history are more likely to develop CIP during ICIs treatment (168, 169). Notably, the past medical history was found to be closely associated with the occurrence of organ-specific irAEs. Recent studies found that patients undergoing cancer immunotherapy with pre-existing interstitial lung disease or asthma experienced a greater incidence of CIP than those without these diseases (168, 169). Similarly, accumulating data highlighted the contribution and prediction of specific gut microbes in the development of ICI-induced intestinal irAEs (170) (**Figure 2**). In addition, a higher baseline absolute eosinophil count (AEC) was observed in patients with CIP than in those without CIP, which could act as a potential biomarker (171) (**Figure 2**). Pavan et al. investigated the predictive value of the neutrophil-to-lymphocyte ratio (NLR) and platelet-to-lymphocyte ratio (PLR) for irAEs in advanced NSCLC treated with ICIs (172). Patients with a lower baseline level of NLR or PLR had a higher risk of irAEs (**Figure 2**). However, results from a retrospective study showed that increased NLR and PLR, and decreased absolute lymphocyte count (ALC) during ICI therapy were associated with the development of CIP in lung cancer patients (149) (**Figure 2**). A study of immune-related cardiotoxicity showed a significant increase in NLR and C-reactive protein (CRP) compared with the baseline value in patients with NSCLC treated with ICIs (173) (**Figure 2**). As a peripheral blood inflammation biomarker, NLR is relatively easy-to-obtain to predict irAEs. However, there is still no consistent point of view. In future, we need a large-sample study on the relationship between baseline NLR levels and irAEs in different cancer types to guide clinical work. Recently, people have developed a new strategy to combine pharmacovigilance and multi-omics data, and a bivariate regression model of LCP1 and ADPGK was established to predict irAEs across different cancer types (174).

## 5 Treatment modalities for irAEs

Treatment of irAEs, which involve multiple organ systems throughout the body and carry a risk of death in severe cases, is critical for cancer patients. For mild to moderate adverse events, suspension or discontinuation of ICIs or treatment with corticosteroid represents the most prevalent and primary treatment modality. A retrospective study of advanced NSCLC showed that glucocorticoids were effective in mitigating mild irAEs and did not affect the efficacy of ICIs (175). Meanwhile, various second-line therapies have been used with some success in patients with corticosteroid-refractory toxicity and serious adverse events. For example, immunoglobulin and plasma replacement therapy can be effective in relieving symptoms of neurogenic immunotoxicity (176, 177). Other immunomodulatory agents also play an important role in the treatment of immunotoxicity. For example, TNF antagonists (infliximab), inosine monophosphate dehydrogenase (IMPDH) inhibitors (mycophenolate), and anti-integrin α4β7 antibodies (vedolizumab) are effective in ameliorating steroid-refractory gastrointestinal toxicity (178–180). As the mechanisms of irAEs have been investigated, cytokine inhibitors are used to ameliorate the symptoms of immunotoxicity. A patient with NSCLC who developed psoriasis flares after treatment with pembrolizumab was treated with an IL-17 inhibitor (secukinumab), which was found to reduce toxicity but did not affect tumor control by pembrolizumab (181). IL-6 inhibitors (tocilizumab) also demonstrated treatment efficiency in patients with irAEs (182). Antibodies against B cells (rituximab) can effectively treat these ICI-induced toxic reactions and some autoimmune toxicities (160, 183). Some other cytokine inhibitors, such as IL-1 inhibitors and IL-23 inhibitors, are effective in relieving clinical symptoms when used in cancer patients presenting with organ-specific irAEs (184). Acupuncture significantly improved neurological symptoms in a patient with advanced lung cancer who developed Guillain-Barre syndrome after receiving immunotherapy (176). There are still many patients with irAEs who still have refractory or immunosuppressive toxicities. A more precise understanding of the pathophysiology of specific irAEs is urgently needed to guide the treatment of severe irAEs.

## 6 Discussion

At present, our understanding of cancer immunotherapy is still at an early stage. Although immunotherapy has significantly improved the survival rate and quality of life in cancer patients as compared to conventional chemotherapy, irAEs caused by immunotherapy pose a great threat to the lives and property of patients. The lack of consensus on the mechanism of irAEs also poses a great challenge to further clinical treatment. Therefore, we still need to learn more about how to apply ICIs more safely and effectively, and how to deal with adverse events caused by ICIs. The mechanism of irAEs and the causes of different adverse reactions caused by different immune drugs need to be further studied. Whether it is possible to screen the high-risk population of irAEs and explore the biomarkers related to the early response and safety of immune drugs remains an essential challenge to address in future. In addition, how to achieve individualized management of immunotoxicity in different organs and at different degrees without compromising immunotherapy is crucial. This article summarized the incidence and mechanism of irAEs, as well as biomarkers to predict immunotoxicity in NSCLC ICI-based immunotherapy. With the further study of predictive markers of immunotoxicity, it was shown that cytokine inhibitors and immunomodulators against autoantibodies have provided a better therapeutic efficacy for organ-specific immunotoxicities. Further research is needed to determine whether organs or patients at risk for irAEs can be pretreated based on immunotoxicity biomarkers.

## Author contributions

WY and SJ designed and conceived the study. XH and LW carried out the literature search and wrote the manuscript draft. BS, JW and JS developed the table. BL and LS prepared the figures. WY critically revised the manuscript. SJ reviewed the manuscript. All authors contributed to the article and approved the submitted version.

## Glossary

**Table:**

ICIs	immune checkpoint inhibitors
PD-1	programmed death-1
PD-L1	programmed death-ligand 1
CTLA-4	cytotoxic T lymphocyte antigen 4
NSCLC	non-small cell lung cancer
irAEs	immune-related adverse events
PFS	progression-free survival
OS	overall survival
FDA	Food and Drug Administration
APCs	antigen-presenting cells
DCs	dendritic cells
Tregs	regulatory T cells
TGF-β	transforming growth factor-β
IL	interleukin
CIP	checkpoint inhibitor pneumonitis
NK	natural killer
LAG-3	lymphocyte activation gene-3
TIGIT	T cell immunoglobulin and ITIM domain
TIM-3	T cell immunoglobulin and mucin-domain containing-3
MHC-II	major histocompatibility complex II
LSECtin	liver sinusoidal endothelial cell lectin
FGL-1	fibrinogen-like protein 1
TILs	tumor-infiltrating lymphocytes
BAL	bronchoalveolar lavage
CRS	cytokine release syndrome
ircAEs	immune-related cutaneous adverse events
GAD65	glutamic acid decarboxylase
IA-2/ICA-512	islet antigen2
ZnT8	zinc transporter8
IAA	insulin autoantibodies
ANA	antinuclear antibody
RF	rheumatoid factor
TG	thyroglobulin
TPO	thyroid peroxidase
CCP	cyclic citrullinated peptide
SP-C	surfactant protein C
AIRE	autoimmune regulator
TERT	telomerase reverse transcriptase
MUC5B	mucin 5B oligomeric mucus/gel-forming
TMB	tumor mutational burden
miR	microRNA
BMI	body mass index
AEC	absolute eosinophil count
NLR	neutrophil to lymphocyte ratio
PLR	platelet to lymphocyte ratio
ALC	absolute lymphocyte count
CRP	C-reactive protein
IMPDH	inosine monophosphate dehydrogenase
TME	tumor microenvironment
COPD	chronic obstructive pulmonary disease
MDSCs	myeloid-derived suppressor cells.
